# A decision tree to improve identification of pathogenic mutations in clinical practice

**DOI:** 10.1186/s12911-020-1060-0

**Published:** 2020-03-10

**Authors:** Priscilla Machado do Nascimento, Inácio Gomes Medeiros, Raul Maia Falcão, Beatriz Stransky, Jorge Estefano Santana de Souza

**Affiliations:** 1 Bioinformatics Postgraduate Program, Metrópole Digital Institute, Federal University of Rio Grande do Norte, Natal, Brazil; 2 Biomedical Engineering Department, Center of Technology, Federal University of Rio Grande do Norte, Natal, Brazil; 3 Bioinformatics Multidisciplinary Environment (BioME), Metrópole Digital Institute, Federal University of Rio Grande do Norte, Natal, Brazil

**Keywords:** Decision tree, VOUS, Pathogenicity, Mutation, Predictor, Precision medicine

## Abstract

**Background:**

A variant of unknown significance (VUS) is a variant form of a gene that has been identified through genetic testing, but whose significance to the organism function is not known. An actual challenge in precision medicine is to precisely identify which detected mutations from a sequencing process have a suitable role in the treatment or diagnosis of a disease. The average accuracy of pathogenicity predictors is 85%. However, there is a significant discordance about the identification of mutational impact and pathogenicity among them. Therefore, manual verification is necessary for confirming the real effect of a mutation in its casuistic.

**Methods:**

In this work, we use variables categorization and selection for building a decision tree model, and later we measure and compare its accuracy with four known mutation predictors and seventeen supervised machine-learning (ML) algorithms.

**Results:**

The results showed that the proposed tree reached the highest precision among all tested variables: 91% for True Neutrals, 8% for False Neutrals, 9% for False Pathogenic, and 92% for True Pathogenic.

**Conclusions:**

The decision tree exceptionally demonstrated high classification precision with cancer data, producing consistently relevant forecasts for the sample tests with an accuracy close to the best ones achieved from supervised ML algorithms. Besides, the decision tree algorithm is easier to apply in clinical practice by non-IT experts. From the cancer research community perspective, this approach can be successfully applied as an alternative for the determination of potential pathogenicity of VOUS.

## Background

The advances of molecular biology and technologies in the last decades were crucial for the generation of large-scale data and the development of “omic” sciences. These data are structured and integrated into new databases that helped the understanding the complexes mechanisms of life and are used to support scientific investigations, either for development of new drugs or disease diagnosis, prevention, and treatments [1]. The databases are being consolidated as a valuable source of information, precise and reliable. Human genetic variations databases highlight among those databases, like the ones based on population frequency of polymorphisms and somatic mutations, many of them focused on cancer [2–7]. Therefore, new data are continually emerging, demanding information extraction in primary analysis, a comparison against the existent knowledge for better exploitation of the acquired evidences, and providing a challenge for data integration [8].

The advent of new generation sequencing (NGS) methods at a low cost set up significant advancements in medicine [9]. The decrease of sequencing price directly influenced the development of precision medicine, making new diagnosis and treatments more precise and individualized [10], and millions of genomes are sequenced every year, either for clinical or research aims [11]. In some cases, the whole genome is sequenced, while others are focused on exome or even in specific genomic regions panel. In this scenario, mutation identification via NGS emerges as a crucial step in the process of disease susceptibility verification and is changing the reality of diagnostics.

A variant calling experiment, which covers mainly protein-coding genome regions, detects up to 20,000 genetic variants in patient’s DNA [12], many of them leading to mutations of diverse types: missense, nonsense, nonstop, frameshift, indel, synonymous, among others. The actual challenge is to precisely identify which mutation plays a critical role in diagnosis or treatments [1]. Variant viewers and computational methods that predict with reasonable accuracy the effects of variants in protein stability can help to identify critical functional mutations [13–16].

Viewer tools like iVariantGuide™ [17], VarSeq™ [18], QueryOR [19], OpenViva (Kroll JE, Nascimento PM, Souza JES, Souza SJ: OpenViva: a variant-calling viewer, unpublished manuscript) are developed to deal with the challenge of information extraction [20], supporting users to access information about detected variants from genetic sequencing, either total or partial. Nevertheless, the amount of information is still very high, and little instructive [21]. Regarding this, specific search filters can be used for easing identification of informative mutations in a case study based on requests by gene, pathogenicity predictors, biochemical characteristics, among other variables. All of this information can be filtered through viewers for extracting only the desired ones for analysis.

After variant annotation, specifics information, namely features, can be aggregated for later use in information filtering, and an important and challenging step is the application of methods and metrics for achieving significant findings [20]. This approach is adopted in OpenViva (Kroll JE, Nascimento PM, Souza JES, Souza SJ: OpenViva: a variant-calling viewer, unpublished manuscript), which provides variant filtering for any feature and considerably accelerates the discovery of relevant mutations. However, there is still much conflicting information that needs to be addressed more carefully.

Although the tools mentioned above provide support for the analysis process, there is still considerable difficulty in integrating biological data. One reason is the great diversity in which databases are distributed [20], which in turn is connected to the problem of backbone design for specific analysis, given the high amount of information for each annotated mutation. Despite the required improvements, information integration can improve the mutation impact predictions and speed up the analysis response time [21].

### Genetic variant impact prediction

Variant impact prediction is based on the identification of base changes, which lead to alteration or loss of the gene function. Variant prediction tools are intensely applied in clinical diagnostic labs to support the mutation effects evaluation in a genetic sequence. However, the methodologies for evaluating the implication of a mutation in pathogenicity vary considerably from a predictor to another [22]. Besides the fact that there is no consensus rule for clinical validation of pathogenicity [23], a suitable choice of a predictor emerges as an essential topic for the clinical area [24].

Three of the most commonly employed predictors in clinical research are Polyphen [14], SIFT [13], and PROVEAN [15]. Polyphen uses structure and evolutionary conservation to predict the impact of amino acid substitution in the structure and function of human proteins [14]. SIFT is based on residues conservation degree, assuming that essential positions in a sequence were conserved during the evolution, so substitutions in these regions have a significant trend to affect proteins function [13]. PROVEAN has a general approach for predicting functional mutation effects in a protein sequence. This predictor scores alterations of a given mutation and verifies the alignment score of sequence like a new metric for predicting harmful effects of the variants [15].

According to Schwarz [24], the average accuracy of these predictors is 85%. Nevertheless, there is a considerable degree of discordance among them regarding the pathogenicity of a mutation, and a manual verification to confirm its real effect becomes necessary. With the development of machine learning computational techniques, specialized in recognizing and associating patterns to a data set, such verification gains the potential to be fully automatized and capable of providing higher correctness rates when analyzing mutations. The Decision Tree is one of these techniques that contribute to achieving this scenario [25].

### Decision tree

Decision Tree is a machine learning technique for data classification in a fixed set of classes and is recommended for problems in which input variables are discrete (have a finite quantity of possible values), and final classification is binary (only two classes) [26]. Assuming that data from variant calling have many ease-discretization features, decision trees become an excellent deterministic model for relevant information search.

As the name suggests, this technique is based on a hierarchical structure of “if-then” rules that produce bifurcations in decision paths (edges), promoting a tree shape. The classification problem, thus, consists of traverse a tree through its edges, in which each node, from the root, executes a test over an input variable. The result indicates which is the next branch of the tree to be traversed, and which node is accessed next. The classification itself is performed when a leaf node, namely, a node with no out-branches, is reached (path ending), and then the class to which input data is better associated is informed. This learning technique has been explored systematically with success in many classification problems in clinical research [27–32]. The present work aims to classify variants in neutral or pathogenic, thus reinforcing the choice of the presented approach (hyperparameter of tree conjecture).

In this work, we describe a decision tree modeling to improve the accuracy of the pathogenicity identification process when the predictors that are regularly used by the scientific community present conflicting results. We expect that this procedure can be used to support the screening of identified mutations, and elucidate a possible role of mutations in the underlying tumorigenesis or response to treatments or diagnosis.

## Methods

### ClinVar

ClinVar is a database designed for propitiating assessment of variants and phenotype relationships in a simplified way [4] and was chosen to evaluate the accuracy of actual predictors and the proposed method. ClinVar was built upon the aggregation of diverse research groups worldwide, to compare and verify the possibility of a consensus over the variant analysis results [33]. Nevertheless, this database describes the impact of only 224,312 mutations and classifies them, as described in Table S1. Facing that, we performed a discretization process over ClinVar according to CLNSIG parameter from this database, considering *neutral variants* the ones marked as CLNSIG 2 or 3, and *pathogenic variants* the ones marked as CLNSIG 4 or 5. ClinVar genome version GRCh38, available in 2017-05-30, was employed for the creation and validation of the model, with a total of 224,312 mutations, from which 31,389 non-synonymous, 13,398 neutral and 17,991 pathogenic, was selected according to the discretization performed. A more recent version of ClinVar, available in 2019-09-23, was used as a future data source for model testing. This new version has 25.052 novel non-synonymous mutations, 8.790 of them are neutral, and 16.082, pathogenic.

The 2017-05-30 version of ClinVar database was used as a learning base that provides a broad knowledge landscape of neutral and pathogenic variants, given the global character that was imprinted during its development, as well as its aim to propose a consensus. For this work, ClinVar was obtained in VCF format, processed in several steps with in-house scripts, and additional data were combined through variant annotation with snpSift and snpEff tools. This base was integrated with databases ExAC [3], 1000genomes [34], HapMap [35], RefSeq [16], among others, and with predictors SIFT [13], Polyphen [14], PROVEAN [15], beyond nine other ones (Table S2), through the support of dbNSFP and in-house scripts.

After annotation with in-house scripts, ClinVar mutations used in training, validation and test were filtered and separated for analysis according to the following criteria: (1) non-synonymous mutations; (2) mutations predicted by SIFT [13], Polyphen [14], and PROVEAN [15]; (3) mutations classified as pathogenic or neutral according to ClinVar. This database was divided into two subsets, one with synonymous variants (for future studies), and another with non-synonymous variants (focus of this study). The latter was submitted to three variant predictors (SIFT [13], Polyphen [14], and PROVEAN [15]), and then split into two new subsets. The first set containing mutations classified as neutral if three predictors classified it so. And the second, mutations classified as pathogenic, in the case of at least one predictor classified it in this way. This process consolidates the first level of the decision tree.

### Evaluation the accuracy of variables

To assess the potential of each classical predictor (SIFT [13], Polyphen [14], PROVEAN [15], MetaSVM [36]), and compare with the effectiveness of seventeen supervised machine-learning (ML) algorithms (described at ML session) with proposed decision tree, we compiled a benchmark to verify the accuracy of each strategy. In this approach, the *Prediction* identifies the predicted mutation as Neutral (N) or Pathogenic (P) and *Clinvar,* identifies Neutral mutation as (0) or Pathogenic mutations as (1). The classification comparison allowed to evaluate the accuracy of diverse strategies as follows: Prediction (N) and Clinvar (0) is True Neutral; Prediction (N) and Clinvar (1) is False Neutral; Prediction (P) and Clinvar (0) is False Pathogenic; Prediction (P) and Clinvar (1) is True Pathogenic. The initial results showed that tree’s conjecture presented an accuracy high enough to be compared with excellent ML algorithms, such as XGBoost and Ada Boost. Conjecture identified Neutral Mutations with False Neutral error rate of 8%; however, False Pathogenic error rate was 9% (Table 1). Features screening was redirected with the intent to diminish the false positives (False Pathogenic error rate).

**Table 1:** Accuracy of proposed model and predictors trained with full ClinVar version 2017-05-30, according to ClinVar version 2019-09-23

Classifier	Accuracy *Mean (± Std. Dev.)	Predictor = N, Clinvar = 0 *Mean (± Std. Dev.)	Predictor = P, Clinvar = 0 *Mean (± Std. Dev.)	Predictor = N, Clinvar = 1 *Mean (± Std. Dev.)	Predictor = P, Clinvar = 1 *Mean (± Std. Dev.)
Extreme Gradient Boosting	93 (0.3)	92 (0.5)	8 (0.5)	7 (0.3)	93 (0.3)
* Proposed Tree	92 (0.3)	91 (0.5)	9 (0.5)	8 (0.3)	92 (0.3)
Random Forest	92 (0.3)	91 (0.5)	9 (0.5)	8 (0.3)	92 (0.3)
Bagging	92 (0.3)	90 (0.5)	10 (0.5)	8 (0.3)	92 (0.3)
K Nearest Neighbors	92 (0.3)	89 (0.5)	11 (0.5)	6 (0.3)	94 (0.3)
Ada Boost	92 (0.3)	93 (0.5)	7 (0.5)	8 (0.3)	92 (0.3)
Extra Trees	91 (0.3)	90 (0.5)	10 (0.5)	8 (0.3)	92 (0.3)
Extra Tree	91 (0.3)	90 (0.5)	10 (0.5)	8 (0.3)	92 (0.3)
Linear Discriminant Analysis	91 (0.3)	88 (0.6)	12 (0.6)	8 (0.3)	92 (0.3)
Support Vector Machines (Linear kernel)	91 (0.3)	86 (0.6)	14 (0.6)	6 (0.3)	94 (0.3)
SKLearn Decision Tree	91 (0.3)	90 (0.5)	10 (0.5)	8 (0.3)	92 (0.3)
Multilayer Perceptron	91 (0.3)	85 (0.6)	15 (0.6)	6 (0.3)	94 (0.3)
Quadratic Discriminant Analysis	91 (0.3)	88 (0.5)	12 (0.5)	8 (0.3)	92 (0.3)
Bernoulli Naive Bayes	91 (0.3)	86 (0.6)	14 (0.6)	7 (0.3)	93 (0.3)
Support Vector Machines (RBF Kernel)	91 (0.3)	86 (0.6)	14 (0.6)	7 (0.3)	93 (0.3)
Logistic Regression	91 (0.3)	86 (0.6)	14 (0.6)	7 (0.3)	93 (0.3)
Gaussian Naive Bayes	90 (0.3)	84 (0.6)	16 (0.6)	6 (0.3)	94 (0.3)
Nu-Support Vector Machines	87 (0.4)	82 (0.6)	18 (0.6)	11 (0.3)	89 (0.3)
PROVEAN	83 (0.4)	75 (0.7)	25 (0.7)	13 (0.4)	87 (0.4)
MetaSVM	81 (0.4)	69 (0.6)	31 (0.6)	10 (0.4)	90 (0.4)
Polyphen	80 (0.4)	82 (0.8)	18 (0.8)	20 (0.3)	80 (0.3)
SIFT	80 (0.4)	77 (0.8)	23 (0.8)	18 (0.4)	82 (0.4)

*Mean and standard were calculated from 1000 random samples, each one with 30% of ClinVar version 2019-09-23

### Features discretization

Based on data integration on ClinVar, we realized the features screening with the potential for partitioning mutations in pathogenic and neutral. After data analysis, we decided to discretize and evaluate ten features as it can be seen from S3 to S12. All discretization was calibrated based on a biological understanding of the variable range, such that a system of binary values replaced attributes of continuous nature.

After each discretization was performed, potential features that could improve the partitioning mutations in pathogenic and neutral were evaluated. Variables describing population allele frequency (ExAC_AF, COMMON), predictors that converge to the same result (Ndamage), mutations that occur in functional domains (Interpro_Domain), changes in biochemical amino acid properties (Transition/transversion sites, Charged/Uncharged, Hydrophobic/Hydrophilic), among others, were analyzed. Once results were obtained in this phase, subsequent levels of decision tree were built. After discretization, six variables were identified as the best features to separate mutation data in neutral and pathogenic: SIFT, Polyphen, PROVEAN, ExAC, NDamage, and COMMON.

### Building the decision tree

**Fig. 1:**
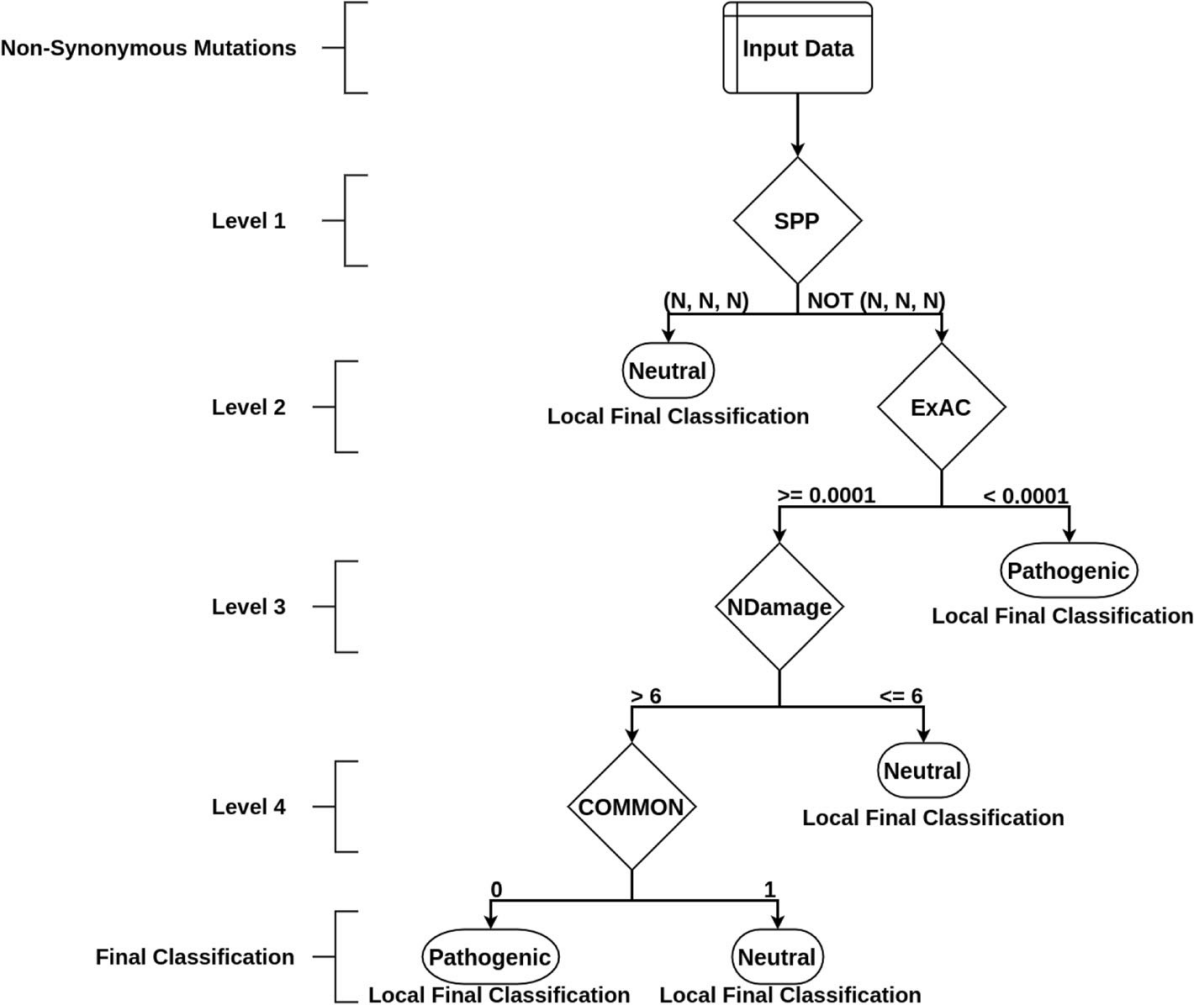
Decision tree obtained after integration and discretization of variables. Level 1: the root node, responsible for receiving input variant, separates the identified mutations in Neutral, if three predictors classify it so (in this case, variant remains not evaluated in future steps), or Pathogenic, if at least one of three predictors classify it so. Level 2: mutations classified as Pathogenic in the previous step are reevaluated according to their allele frequency in ExAC database, being reclassified as Neutral for mutations with allele frequency higher than 0.0001 (remains not evaluated in future steps), or maintained as Pathogenic, for mutations with allele frequency less than 0.0001. Level 3: Mutations previously classified as Pathogenic are reevaluated according to the number of predictors that converge to the same result. Mutations are reclassified as Neutral if identified as pathogenic by less than five predictors (remains not evaluated in future steps), or maintained as Pathogenic if identified as so by five to nine predictors. Level 4: Mutations previously classified as Pathogenic are reevaluated according to the COMMON variable from 1000genomes, being reclassified as Neutral for mutations with allele frequency higher than 0.0001, or maintained as Pathogenic, for mutations with allele frequency less than 0.0001

After the identification of variables and the respective True-False discretization processes, representing mutation characteristics, the decision tree building was performed. A greedy algorithm was employed for evaluating the potential features for partitioning mutations in pathogenic and neutral in each tree level, selecting those that provide the highest degrees of accuracy (according to ClinVar annotation). When the tree level converged to a local optima feature (best partitioning results), a next level was established, and all remaining features were tested in brute force mode again until a final shaped tree was designed (Fig. 1).

The tree was designed in a greedy way - each level is based on discretized variables and best separation accuracy (neutral/pathogenic), using a training and a validation datasets. The tree topology is illustrated in Fig. 1, composed of four nodes that evaluate six variables: SPP,^1^ ExAC, NDamage, and COMMON.

### Variable discretization and pathogenicity features discovery

After discretize ten variables - ExAC_AF, COMMON, NDamage, Interpro_Domain, Transition/transversion sites, Charged/uncharged, Hydrophobic/hydrophilic, Essential/non-essential, Initial/not initial exon and PPI (Tables S3, S4, S5, S6, S7, S8, S9, S10, S11 and S12, respectively), four of them showed a better segregation among categorizations (ExAC_AF, COMMON, NDamage, Interpro_Domain - Tables S3, S4, S5 and S6). No difference was observed for other variables frequencies (Transition/transversion sites, Charged/uncharged, Hydrophobic/hydrophilic, Essential/non-essential, Initial/not initial exon, and PPI), and they were not used in the decision tree (Tables S7, S8, S9, S10, S11 and S12).

### Machine learning algorithms

For evaluating the potential of proposed model, a comparison was realized with following Machine Learning (ML) algorithms: Extreme Gradient Boosting, Ada Boost, K Nearest Neighbors, Bagging, Random Forest, Extra Trees, SKLearn Decision Tree, Linear Discriminant Analysis, Linear Support Vector Machines with RBF and Linear Kernels, Nu-Support Vector Classification, Logistic Regression, Multilayer Perceptron (1 hidden-layer of one hundred neurons), Quadratic Discriminant Analysis, Bernoulli Naive Bayes, and Gaussian Naive Bayes. It was used the implementation provided by scikit-learn^2^ library, with except for Extreme Gradient Boosting, for which implementation from xgboost^3^ library was used. SKLearn Decision Tree, Extreme Gradient Boosting, Ada Boost, Bagging, Random Forest, and Extra Trees are ensemble methods that construct and combine a set of decision trees for classifying data. K Nearest Neighbours classifies data based on their Euclidean distances and vicinity. Linear Discriminant Analysis, Linear Support Vector Machines with RBF and Linear Kernels, Nu-Support Vector Classification, and Quadratic Discriminant Analysis seek to perform classification by dividing data representation space in hyperplanes. Logistic Regression and Multilayer Perceptron optimize predefined sets of equations for classifying data into their respective classes, and Bernoulli and Gaussian Naive Bayes uses Bayes theorem in a probabilistic fashion for classification.

### Cross-validation

10-fold Cross-Validation was performed to test the potential of the proposed model and find the best training and testing conjecture. Pathogenic and Neutral mutations data from the 2017-05-30 version of the ClinVar database were each divided into 10 folds. Each pair of folds from pathogenic and neutral mutations are selected in a 10-step iterative process to form a test dataset while remaining folds are joined together to form a training dataset.

In each step, ML algorithms are trained and then classify all mutations from the test dataset. The following metrics are then calculated: (a) Accuracy, calculated as (True Neutrals + True Pathogenic)/(True Neutrals + False Neutrals + True Pathogenic + False Pathogenic); (b) Sensitivity (Predictor = N, Clinvar = 0), calculated as True Neutrals/(True Neutrals + False Neutrals); (c) Type I Error (Predictor = P, Clinvar = 0), calculated as False Neutrals/(True Neutrals + False Neutrals); (d) Specificity (Predictor = P, Clinvar = 1), calculated as True Pathogenic/(True Pathogenic + False Pathogenic); and (e) Type II Error (Predictor = N, Clinvar = 1), calculated as False Pathogenic/(True Pathogenic + False Pathogenic). The best conjecture, defined as the pair of training and test datasets that yielded the highest accuracy, was selected for each algorithm after the execution of Cross-Validation.

### Decision tree, classical predictors and ML algorithms tests

To evaluate the efficiency of the proposed tree, classical predictors and ML algorithms, they were trained with the entire ClinVar 2017-05-30 dataset. Subsequently they were submitted to testing using Monte Carlo simulation with the ClinVar 2019-09-23 dataset, in an iterative process of a thousand steps. In every iteration, a sample containing 30% mutations from ClinVar was selected randomly, evaluated by each classifier, and rates were then calculated from these classifications, with the mean and standard deviation.

The best performance of each ML algorithm was established by the cross-validation test. Again they were submitted to testing using Monte Carlo simulation with the ClinVar 2019-09-23 dataset.

### Distribution of variables

Distribution of neutral and pathogenic mutations of variables used in the proposed model for the best conjecture of training and validation, so as for validation sets, are shown in Table S13. These same distributions were calculated for each two-by-two combination of the proposed model variables (neutral-neutral, neutral-pathogenic, pathogenic-neutral, and pathogenic-pathogenic), as shown in Figure S1.

## Results

### Accuracy of classifiers

To validate the proposed method, we compared the result obtained by the decision tree with the ones obtained by the four classical pathogenicity predictors, SIFT [13], Polyphen2 [14], PROVEAN [15] and MetaSVM [36], and the seventeen ML algorithms described in Methods, evaluated over same data. These algorithms were trained on all ClinVar data (version 2017-05-30), and tested using a Monte Carlo simulation with ClinVar version 2019-09-23. The mean and standard deviation of each fold are available in Table 1. Also, we performed 10-fold cross-validation with mutations data from the 2017-05-30 version of the ClinVar database, and the results (in terms of the mean and standard deviation of each fold) are available in Table S14.

The results showed that the decision tree reached higher precision in every tested variable: 91% for True Neutral, 8% for False Neutral, 9% for False Pathogenic, and 92% for True Pathogenic. For example, SIFT results were: 77% for True Neutral, 18% for False Neutral, 23% for False Pathogenic, and 82% for True Pathogenic; and MetaSVM results were: 69% for True Neutral, 10% for False Neutral, 31% for False Pathogenic, and 90% for True Pathogenic (Table 1). As mentioned previously, the decision tree demonstrated higher precision in the classification of these data, consistently producing relevant predictions for realized tests.

It can be noticed from Table 1 and S14 that all predictors achieve statistically the same performance in both ClinVar versions (considering mean and standard deviations). The proposed method has a mean accuracy of 91 (±0.1)% in 10-fold cross-validation results, and a mean accuracy of 92 (±0.3)% in Monte Carlo simulation, with overlap confidence intervals. This behavior is also seen from best ML algorithms, (for example, Extreme Gradient Boosting has mean accuracy of 92 (±0.0)% in cross-validation, and 93 (±0.3)% in Monte Carlo simulation) and classical predictors (for example, PROVEAN has mean accuracy of 80 (±0.1)% in cross-validation, and 83 (±0.4)% in Monte Carlo simulation).

All classical predictors obtained the worst accuracies in both test scenarios: MetaSVM had a mean accuracy of 82 and 81% in Cross-Validation and Monte Carlo simulation, respectively; PROVEAN, 80, and 83%; SIFT, 78, and 80%; and Polyphen, 77, and 80%. These measures are below the ones reported in the literature [24].

Our proposed method was ranked between the six best positions in accuracy evaluation from both analyses (Table 1 and S14), with a difference of only 1% from the first two (Extreme Gradient Boosting and Ada Boost), and a mean difference of 9% to classical predictors. These results show the competitive strength of the model, considering both the powerful ML algorithms and traditional predictors used in clinical research for pathogenicity in VOUS.

### Variables distribution

The results in Figure S1 show that when variables are combined, possible classification errors that could be made by one is compensated by another. For example, 45.3% of ClinVar neutral mutations (2017-05-30 version) are classified as neutral by the SPP variable and as pathogenic by the ExAC variable. Therefore, in the combination SPP-ExAC, the first variable prevents 45.3% of mutations from being misclassified by the second. Similarly, 21.6% of pathogenic mutations have the neutral-pathogenic combination of the ExAC-NDamage pair, making it possible to state that the second variable compensate in 21.6% the wrong classifications of the first. Such behavior suggest a classification complementarity among tree variables, and when using them together, achieves a higher accuracy in relation to their application alone.

### Variable correlations

The φ coefficient, as described in [37], was used to verify the degree of correlation of the four variables according to ClinVar (2017-05-30 version). Table S15 presents the results obtained for each two-by-two combination of variables. It is important to note that tree variables do not present a strong correlation with each other except for the ExAC-COMMON pair (− 0.77) as shown above.

### Complementarity of variables and variants of the proposed model

Eleven new tree topologies were produced from rearrangements of the obtained topology. It is important to note here that although the ExAC and COMMON population variables have high discriminatory power (ExAC: 95% pathogenic, 81% neutral, COMMON: 98% pathogenic, 58% neutral) and represent a large amount of information extracted from the ExAC and 1000 genomes, respectively, they do not present a significant representativeness of populations with low sequencing level. In our strategy, variants with low population frequency are treated as pathogenic. Neutral high frequency mutations in populations not yet sequenced, however, would not have been described in these banks and would therefore be misclassified as pathogenic.

As this interpretation could lead to high error rates in future data, we discarded the trees that presented these variables in the root node, limiting this position to the variables SPP and NDamage. This resulted in twelve possible topologies (the eleven cited plus our suggested model). The accuracy, false positive rate, and false negative rate on ClinVar (version 2017-05-30) were calculated for each level of the trees, as shown in Table 2.

**Table 2:** Accuracies at each level of constructed tree topologies variants from our proposed model, according to ClinVar (version 2017-05-30)

	Accuracy (%)				FPR (%)				FNR (%)			
Topology	Lv. 1	Lv. 2	Lv. 3	Lv. 4	Lv. 1	Lv. 2	Lv. 3	Lv. 4	Lv. 1	Lv. 2	Lv. 3	Lv. 4
**SPP-ExAC-NDamage-COMMON (Proposed Tree)**	74	91	90	91	30	8	11	9	10	11	9	9
SPP-ExAC-COMMON-NDamage	74	91	87	91	30	8	16	9	10	11	8	9
SPP-NDamage-ExAC-COMMON	74	78	79	79	30	7	2	4	10	32	33	32
SPP-NDamage-COMMON-ExAC	74	78	79	79	30	7	4	2	10	32	32	33
SPP-COMMON-ExAC-NDamage	74	87	91	91	30	15	8	9	10	8	11	9
SPP-COMMON-NDamage-ExAC	74	87	79	79	30	15	4	2	10	8	32	33
NDamage-SPP-ExAC-COMMON	78	78	79	79	7	7	2	4	32	32	33	32
NDamage-SPP-COMMON-ExAC	78	78	79	79	7	7	4	2	32	32	32	33
NDamage-ExAC-SPP-COMMON	78	79	78	79	7	2	7	4	32	33	32	32
NDamage-ExAC-COMMON-SPP	78	79	79	79	7	2	4	4	32	33	32	32
NDamage-COMMON-SPP-ExAC	78	79	79	79	7	4	4	2	32	32	32	33
NDamage-COMMON-ExAC-SPP	78	79	79	79	7	4	2	4	32	32	33	32

## Discussion

### Sequencing in clinical genomics

Bioinformatics provides an essential bridge between science and delivery of relevant information for genomic medicine in practical clinics [36, 38]. Only in England, after 100.000 genomes project announcement [39], thirteen genomic medicine centers were opened in 2014 [36]. This leverage stands out due to the ever-developing nature of health and the significant advancements in omic sciences in the last 15 years [40]. Bioinformatics had a fundamental role in the genomic revolution in the last two decades [10], but still has a lot to accomplish and contribute.

The large volume of data in genomic sequencing is such that a huge task for any research center is to identify and categorize automatically all detected mutations without an analyst or curator intervention. Aggregate data and discoveries from many known sources can speed up the process by which pathogenic genetic mutations are identified, allowing a personalized treatment approach for each patient [41]. Databases like DECIPHER [42], COSMIC [43], HGMD [44], OMIN [45], ClinVar [4], and NCBI dbGaP [46], can help to link genetic mutations to known phenotypes. However, even these databases are incipient, and cannot always be useful for genes and mutations limited studied, or even identified mutations in poorly studied populations [47].

The pathogenicity predictors seem to be an excellent option for the determination of pathogenicity potential of a variant of unknown meaning (VOUS, Variant of Unknown Significance). This is a hot and active research field: there are notoriously known predictors, such as SIFT [13], Polyphen2 [14], PROVEAN [15], MetaSVM [36], among others. However, conflicting predictions among predictors and the average accuracy of 85% of these strategies indicate that there is space for seeking better accuracy via an automated process of pathogenicity determination [47].

We described in this work the modeling of a decision tree and the discretization of variables (attributes from database integration) to improve the average accuracy of actual pathogenicity predictors. In our tests, when results from decision tree were compared to predictors, decision tree reached higher precision in every tested variable: 91% for True neutral, 9% for False neutral, 9% for False Pathogenic, and 91% for True Pathogenic (Table S14). When we compared the proposed model with ML algorithms (Table 1), we verified that its performance is comparable to the best ones, because it has an accuracy of 92%, slightly lower or equal than accuracies from Extreme Gradient Boosting and Ada Boost, respectively, 93 and 92%. These rates are superior even from a meta-predictor that unites results from various predictors, the MetaSVM [36], whose results were: 69% for True neutral, 10% for False neutral, 31% for False Pathogenic, and 90% for True Pathogenic (Table 1). Decision tree exceptionally demonstrated high precision in the classification of these data, consistently producing relevant previsions for realized tests, which makes it an excellent option for the determination of pathogenicity potential of VOUS.

We also reevaluate the pathogenicity prediction potential of actual predictors, to highlight individual prediction potential of tested variables (True Neutral, False Neutral, False Pathogenic, and True Pathogenic) for each predictor (Table 1). MetaSVM [36] has the highest identification rate of True Pathogenic (90%), while Polyphen2 [14] has the highest identification rate of True Neutral (82%). MetaSVM has the lowest rate of False Neutral (10%), while Polyphen2 [14] has the lowest rate of False Pathogenic (18%).

### Decision tree-based predictors vs. weighting-based predictors

Weighting-based predictors, as the name suggests, initially perform a weighted sum up classifying input data, submitting the result to an activation function, destined to proper classification [48]. Decision Tree-based techniques, on the other hand, organize datum variables in a hierarchical data structure (tree), where each node corresponds to an “if-then” execution rule over a specific variable of datum [26]. Given this definition, it can be assumed that variables have a meaningful contribution to the final result in weighting algorithms. Moreover, alterations in weights significantly impact the classification result. Thus, every variable must be considered during the analysis, which implies that weighting algorithms have few or no flexibility in their design, because of inherently sensibility to value changes of weighting [48].

Diversely, the decision tree-based techniques provide different classification methods, in which not all variables are considered in every case, making this approach much more feasible to classification problems, especially with big data. In the proposed tree, if the result of SPP node considers that analyzed mutation is neutral, it is not necessary to consider remaining variables. Therefore, this approach is not only flexible but also efficient, once information volume to be processed is reduced.

Another inherent advantage to decision trees is the ease of manipulating it, adding or removing nodes (rules) without necessarily affect its global performance [49]. In the SPP node example, if a new node were added in one branch of the tree, the classification processes that go from the branch with a new rule would not affect tree branches where the rule was not inserted, which implies in performance preservation of that branch. This same inclusion in a weighting-based algorithm would influence in its whole performance raising a real possibility of diminishing the efficiency in the classification of a set of cases previously correct classified, for example, the comparison of prediction performance between Polyphen2 [14] and MetaSVM [36] (Table 1).

It is essential to highlight that the inclusion of a new classifying feature in weighting-based prediction algorithm would imply in defining associated weight to that feature so as complete re-balancing of the sum should be done. Considering that weighting predictors SIFT [13] and PROVEAN [15] do not consider allowing the possibility of including new classifying features and add new features to them is an infeasible task. It causes a negative impact because a significant part of actual knowledge about mutations came from projects made after their implementations and publications, i.e., ExAC [3], 1000genomes [34], and COSMIC [43], so these predictors can be considered outdated. Therefore, the proposed prediction method is a promising complementation to traditional mutation prediction tools, since it presents excellent flexibility in manipulating decision rules, as a way to enhance its predictive power. This improvement was demonstrated in the correct classification percentages of the proposed tree, that overcomes other traditional tools (Table 1).

### Proposed tree versus ML algorithms

All machine-learning algorithms are presented as a black box (in general, the user only executes them without interfering with their functioning or understanding their execution flow). The lack of this transparency, combined with the high computational cost required for its use, makes it difficult, in many cases, its direct use in clinical practice. The proposed decision tree, in turn, is based on biological precepts and simple data analysis to make a decision. The simplicity of data processing, coupled with accuracy, facilitates its application in clinical practice by non-IT professionals.

The proposed model is organized from data that can be easily obtained through web tools. For example, a physician, from a sequencing clinical report, could collect through web tools information on the variants in question, such as allelic frequency (via ExAC and COMMON), as well as submitting them to online versions of the classical predictors used in this work. With the resulting data, he could analyze them under the light of the proposed decision tree to determine which of these variants are pathogenic, since the model proposed here boils down to comparing the values ​​of such data with other values ​​that are capable of identifying a variant as pathogenic or benign.

Thus, the model presented in this paper has the potential for immediate practical application, compared to more complex models provided by other machine-learning algorithms. In this sense, it is intended, as future objective, to implement a user-friendly tool that automates the collection of this data, as well as execute the proposed model on them.

### Combinations of predictors

Because of the diversity of algorithms classes destined to information prediction, it is natural that two different predictors not always provide the same classification result for the same input data. In machine learning, ensembles of classifiers term are given to the attempt to combine the outputs of one or more prediction algorithms. As reported by various works [50–54], this technique produces better results if compared to the use of individual predictors. Two nodes from the proposed prediction method in this work are ensembles. The first one is the root, which combines predictions of SIFT [13], Polyphen2 [14], and PROVEAN [15]. The second is Ndamage, an ensemble of nine mutation classifiers. One parameter that must be taken into consideration when building an ensemble is the number of employed classifiers because a high quantity can negatively impact the predictor’s accuracy [55]. Although there are two ensembles of compound-classifiers, it did not affect the overall tree accuracy, and tree conjecture contribute to a considerable decrease in false negatives rate to 9%, both in Cross-Validation and Monte Carlo simulations (Tables S14 and 1, respectively), and was fixed in distinct positions of the tree.

Another factor that contributed to such decision was the goal of preserving tree branches that were already providing positive results. As previously mentioned, the SPP ensemble as root node contributed to the proposed tree has low False Neutral rates. Although False Pathogenic rates are still high (56%), tree flexibility character independently allowed the decrease of it in other branches, without affecting meaningfully True Pathogenic. Based on this, the remaining of the proposed tree focused on filtering neutral mutations, making it possible to classify with assurance a mutation as Pathogenic at the end of the process. As new neutral classification branches that contribute to diminishing False Pathogenic were discovery through that strategy, they were preserved, and new branches for treating possible pathogenic mutations were defined.

## Conclusions

This paper showed that application of Machine Learning on the available high-throughput data is a valuable tool for biomedical research, and provides a significant case study to other researchers that are dealing with similar discovery challenges. A new method of pathogenicity classification of the VOUS mutations is proposed in this work. The decision tree, modeled as a classification process, was based on the evaluation of statistical properties of input classifying variables obtained through data integration. Besides, we present a comparative benchmark of the accuracy of the proposed method concerning other prediction tools.

The proposed method was validated with the ClinVar database [4], and its performance was compared to predictors SIFT [13], Polyphen2 [14], PROVEAN [15] and MetaSVM [36], showing to be more accurate regarding the correct identification of pathogenic variants, with the lowest False Pathogenic rate. This result is meaningful because the classification of the mutation potential plays a crucial role in response to treatment and diagnosis. When compared to seventeen ML algorithms, the proposed method kept as one of the bests in terms of accuracy, showing its high prediction potential.

For future works, it is intended to explore other variables not employed in this work for refining the built tree, seeking to improve the method performance. Moreover, it is also aimed to study and develop generator algorithms of mutation prediction trees with optimal performance, employing evolutionary computation techniques. This methodology is being explored intensively in decision trees design [56], and gene expression studies [57].

It is important to note that this work is focused on non-synonymous mutations and VOUS pathogenicity classification, aiming to identify potential deleterious variances. However, the features discretization approach proposed in this work enables the construction of other trees with different proposals, such as the search for variants involved in genetic syndromes, just requiring the input of adequate characteristic variables. Finally, when results from tests over the decision tree were compared to the ones from predictors with the same data, the decision tree reached the highest precision among all tested variables. The classification of those data, whereas consistently provide relevant previsions for realized tests, establish an alternative and more precise option for determination of pathogenicity potential of VOUS for the cancer research community.

## Supplementary information

**Additional file 1: Table S1.** Annotation of clinical variants, using terms from ClinVar VCFs, according to the map below. **Table S2.** Predictors contained in NDamage. **Table S3.** Discretization of ExAC_AF variable: allele frequency of variants based on all samples from ExAC. **Table S4.** Discretization of COMMON variable, based on 1000genomes database. **Table S5.** Discretization of NDamage variable: number of predictors that point out a variant as pathogenic. **Table S6.** Discretization of Interpro_Domain variable: functional domain or site associated to mutation. **Table S7.** Discretization of Transition/transversion variable, based on nucleotide transversions or transitions. **Table S8.** Discretization of Charged/uncharged variable. **Table S9.** Discretization of Hydrophobic/hydrophilic variable. **Table S10.** Discretization of Essential/non-essential variable. **Table S11.** Discretization of Initial/not initial exon variable: mutations affecting gene start are more impactant. **Table S12.** Discretization of PPI variable. **Table S13.** Distribution of neutral and pathogenic mutations for each variable of the proposed model (in percent fraction number), in Best Conjecture of Training, Validation, and Testing, so as in 1000-step Monte Carlo simulation dataset. **Table S14.** Accuracy of predictors and proposed model in 10-fold Cross-Validation process, according to ClinVar version 2017-05-30. Mean and standard were calculated from ten steps of 10-fold Cross Validation. **Table S15.** Coefficient φ for the two-by-two variable combinations of the proposed model. **Figure S1.** Distribution of neutral and pathogenic mutations of variables used in proposed tree, according to ClinVar version 2017-05-30. In tables’ columns and row names, 0 indicates the mutations classified by a variable as neutral, and 1, as pathogenic. In tables’ cells, numbers at left of the slash indicates the percentage of neutral mutations for each classification combination of each variables pair, and numbers at right of the slash, the percentage of pathogenic mutations.

## Data Availability

The datasets filtered and/or categorized during the current study are available in our repository, at http://www.bioinformatics-brazil.org/TreeSPP/clinvar.zip.

## Abbreviations

ML: Machine learning
PPI: Protein-protein interaction
SPP: Ensemble of variables SIFT, PROVEAN, and Polyphen
VCF: Variant call format
VOUS: Variant of unknown significance

## Footnotes

1 Ensemble of variables SIFT, PROVEAN, and Polyphen.

2 https://scikit-learn.org

3 https://xgboost.readthedocs.io
