# Supplementary material for: A decision tree to improve identification of pathogenic mutations in clinical practice
Source: BMC Med Inform Decis Mak. 2020 Mar 10;20:52. doi: 10.1186/s12911-020-1060-0 (PMC7063785; doi:10.1186/s12911-020-1060-0)
Supplement: Supplementary file 1 — Additional file 1: Table S1. Annotation of clinical variants, using terms from ClinVar VCFs, according to the map below. Table S2. Predictors contained in NDamage. Table S3. Discretization of ExAC_AF variable: allele frequency of variants based on all samples from ExAC. Table S4. Discretization of COMMON variable, based on 1000genomes database. Table S5. Discretization of NDamage variable: number of predictors that point out a variant as pathogenic. Table S6. Discretization of Interpro_Domain variable: functional domain or site associated to mutation. Table S7. Discretization of Transition/transversion variable, based on nucleotide transversions or transitions. Table S8. Discretization of Charged/uncharged variable. Table S9. Discretization of Hydrophobic/hydrophilic variable. Table S10. Discretization of Essential/non-essential variable. Table S11. Discretization of Initial/not initial exon variable: mutations affecting gene start are more impactant. Table S12. Discretization of PPI variable. Table S13. Distribution of neutral and pathogenic mutations for each variable of the proposed model (in percent fraction number), in Best Conjecture of Training, Validation, and Testing, so as in 1000-step Monte Carlo simulation dataset. Table S14. Accuracy of predictors and proposed model in 10-fold Cross-Validation process, according to ClinVar version 2017-05-30. Mean and standard were calculated from ten steps of 10-fold Cross Validation. Table S15. Coefficient φ for the two-by-two variable combinations of the proposed model. Figure S1. Distribution of neutral and pathogenic mutations of variables used in proposed tree, according to ClinVar version 2017-05-30. In tables’ columns and row names, 0 indicates the mutations classified by a variable as neutral, and 1, as pathogenic. In tables’ cells, numbers at left of the slash indicates the percentage of neutral mutations for each classification combination of each variables pair, and numbers at right of the slash, the percent [file 12911_2020_1060_MOESM1_ESM.docx]

Table S1 - Annotation of clinical variants, using terms from ClinVar VCFs, according to the map below.

| **ASN.1 Terms** | **ClinVar** |
| --- | --- |
| 0 – Unknown | Uncertain significance |
| 1 – Untested | not provided (includes the cases where data are not available or unknown) |
| 2 – Non-Pathogenic | Benign |
| 3 – Probable-Non-Pathogenic | Likely benign |
| 4 – Probable-Pathogenic | Likely pathogenic |
| 5 – Pathogenic | Pathogenic |
| 6 – Drug-Response | Drug Response |
| 7 – Histocompatibility | Histocompatibility |
| 255 – Other | Other |
|  | Confers Sensitivity, Risk Factor, Association, Protective, Affects |

Source: <https://www.ncbi.nlm.nih.gov/clinvar/docs/clinsig/>

Table S2 - Predictors contained in NDamage.

| **Predictor** | **Source** |
| --- | --- |
| FATHMM | [http://fathmm.biocompute.org.uk](http://fathmm.biocompute.org.uk/) |
| LRT | <http://www.genetics.wustl.edu/jflab/lrt_query.html> |
| MetaLR | <http://annovar.openbioinformatics.org/en/latest/user-guide/filter/#-metalr-annotation> |
| MetaSVM | <https://github.com/jjh0925/metaSVM> |
| MutationAssessor | <http://mutationassessor.org/> |
| MutationTaster | <http://www.mutationtaster.org/> |
| Polyphen2_HDIV | <http://genetics.bwh.harvard.edu/pph2/> |
| PROVEAN | <http://provean.jcvi.org/index.php> |
| SIFT | <http://sift.bii.a-star.edu.sg/> |

Table S3 - Discretization of ExAC_AF variable: allele frequency of variants based on all samples from ExAC.

| ClinVar | #Mutations | % | ExAC_AF |
| --- | --- | --- | --- |
| 0 | 6067 | 81.12 | Freq >= 0.0001 |
| 0 | 1412 | 18.88 | Freq < 0.0001 |
| 1 | 869 | 5.00 | Freq >= 0.0001 |
| 1 | 16495 | 95.00 | Freq < 0.0001 |

The discretization process of this feature was grouping in class Freq >= 0.0001 registers with values equal to or higher than, and in class Freq < 0.0001 missing values or the ones that were below 0.0001.

Table S4 - Discretization of COMMON variable, based on 1000genomes database.

| ClinVar | #Mutations | % | dbSNP.COMMON |
| --- | --- | --- | --- |
| 0 | 3120 | 41.71 | 0 |
| 0 | 4359 | 58.29 | 1 |
| 1 | 17089 | 98.41 | 0 |
| 1 | 275 | 1.59 | 1 |

If the frequency of least frequently allele is higher or equal than 0,0001 in at least one population from 1000 genomes and have the contribution of at least two individuals from different families for this allele, then value 1 is attributed to the variable, otherwise value 0 is so. The discretization of this feature defines that mutations whose COMMON values are equal to 1 are grouped in class 1, and mutations with a missing value for COMMON and value equal 0 are grouped in class 0.

Table S5 - Discretization of NDamage variable: number of predictors that point out a variant as pathogenic.

| ClinVar | #Mutations | % | NDamage |
| --- | --- | --- | --- |
| 0 | 861 | 11.51 | >= 6 |
| 0 | 6618 | 88.49 | < 6 |
| 1 | 12043 | 69.35 | >= 6 |
| 1 | 5321 | 30.64 | < 6 |

This number varies from 0 to 9. Class >=6 groups mutations with at least five predictors that classify them as pathogenic, otherwise they are grouped in class <6.

Table S6 - Discretization of Interpro_Domain variable: functional domain or site associated to mutation.

| ClinVar | #Mutations | % | Interpro Domain |
| --- | --- | --- | --- |
| 0 | 3566 | 47.68 | 0 |
| 0 | 3913 | 52.32 | 1 |
| 1 | 12785 | 73.63 | 0 |
| 1 | 4579 | 26.37 | 1 |

The annotation comes from the Interpro database. Discretization of this variable is organized in two classes: if a mutation occurs in an Interpro domain, it is grouped in class 1; otherwise, class 0.

Table S7 - Discretization of Transition/transversion variable, based on nucleotide transversions or transitions.

| ClinVar | #Mutations | % | Transition/transversion |
| --- | --- | --- | --- |
| 0 | 5437 | 72.69 | 0 |
| 0 | 2042 | 27.31 | 1 |
| 1 | 11941 | 68.76 | 0 |
| 1 | 5423 | 31.24 | 1 |

This variable describes whether substitution is a transversion (class 1), or a transition (class 0).

Table S8 - Discretization of Charged/uncharged variable.

| ClinVar | #Mutations | % | Charged/uncharged |
| --- | --- | --- | --- |
| 0 | 4314 | 57.68 | 0 |
| 0 | 3165 | 42.32 | 1 |
| 1 | 8781 | 50.57 | 0 |
| 1 | 8583 | 49.43 | 1 |

The amino acid charge is associated with its polarity. If an amino acid is apolar, so its charge is neutral. In the case of it be polar, either its charge is positive or negative; either aminoacid is partially charged. If amino acid change altered totally or partially its charge, then its class is 1; otherwise, it is 0.

Table S9 - Discretization of Hydrophobic/hydrophilic variable.

| ClinVar | #Mutations | % | Hydrophobic/hydrophilic |
| --- | --- | --- | --- |
| 0 | 4300 | 57.49 | 0 |
| 0 | 3179 | 42.51 | 1 |
| 1 | 8478 | 48.83 | 0 |
| 1 | 8886 | 51.17 | 1 |

Protein nucleus tends to contain more apolar amino acids, without charge, because its surface is in the water interface. Changes in this charge have a direct impact on the structure and function of a protein. This variable describes whether or not there was charge change (polar or apolar). If there was polarity change, so class 1; otherwise, class 0.

Table S10 - Discretization of Essential/non-essential variable.

| ClinVar | #Mutations | % | Essential/non-essential |
| --- | --- | --- | --- |
| 0 | 4390 | 58.70 | 0 |
| 0 | 3089 | 41.30 | 1 |
| 1 | 9844 | 56.70 | 0 |
| 1 | 7520 | 43.30 | 1 |

Amino acids are classified as essential and nonessential. The latter are produced by own organism. Thus, a substitution that changes aminoacid classification from nonessential to essential can have a relevant impact on its production. The following discretization rule was applied: if there was a change in aminoacid classification, then attribute to class 1; otherwise, to class 0.

Table S11 - Discretization of Initial/not initial exon variable: mutations affecting gene start are more impactant.

| ClinVar | #Mutations | % | Initial/not initial exon |
| --- | --- | --- | --- |
| 0 | 843 | 11.27 | 0 |
| 0 | 6636 | 88.73 | 1 |
| 1 | 1312 | 7.55 | 0 |
| 1 | 16052 | 92.45 | 1 |

This kind of event can take to a gain or even loss of function of a gene. Discretization was performed according to the following rule: if a mutation occurs in less than 10% of the initial part of gene cds, namely, initial region, attribute to class 0; otherwise, class 1.

Table S12 - Discretization of PPI variable.

| ClinVar | #Mutations | % | PPI |
| --- | --- | --- | --- |
| 0 | 5901 | 78.90 | >= 5 |
| 0 | 1578 | 21.10 | < 5 |
| 1 | 14468 | 83.32 | >= 5 |
| 1 | 2896 | 16.68 | < 5 |

Depending on the number of interactions with other proteins, a mutation in a gene can imply a cascade effect, so affecting other genes in an interaction network. Discretization classes were created in the following way: mutations in gene with PPI value equal to or greater than 5 form class >=5; values whose PPI are lesser than 5 are grouped in class <5.

Table S13 - Distribution of neutral and pathogenic mutations for each variable of the proposed model (in percent fraction number), in Best Conjecture of Training, Validation, and Testing, so as in 1000-step Monte Carlo simulation dataset.

|  | **Best Conjecture**  **of Training** | | **Best Conjecture**  **of Validation** | | | **1000-step Monte Carlo**  **simulation dataset** | | |
| --- | --- | --- | --- | --- | --- | --- | --- | --- |
|  | **Neutral (%)** | **Pathogenic (%)** | | **Neutral (%)** | **Pathogenic (%)** | | **Neutral**  **(% ± Std. Dev.)** | **Pathogenic**  **(% ± Std. Dev.)** |
| SIFT = N | 62 | 11 | | 66 | 10 | | 61 (0.8) | 10 (0.4) |
| SIFT = P | 38 | 89 | | 34 | 90 | | 39 (0.8) | 90 (0.4) |
| Polyphen = N | 57 | 7 | | 57 | 9 | | 54 (0.9) | 6 (0.3) |
| Polyphen = P | 43 | 93 | | 43 | 91 | | 45 (0.9) | 94 (0.3) |
| PROVEAN = N | 74 | 16 | | 79 | 16 | | 75 (0.8) | 13 (0.4) |
| PROVEAN = P | 26 | 84 | | 21 | 84 | | 24 (0.8) | 87 (0.4) |
| ExAC >= 0.0001 | 81 | 6 | | 84 | 5 | | 73 (0.8) | 2 (0.1) |
| ExAC < 0.0001 | 19 | 94 | | 16 | 96 | | 26 (0.8) | 98 (0.1) |
| NDamage <= 6 | 93 | 33 | | 94 | 34 | | 92 (0.5) | 30 (0.6) |
| NDamage > 6 | 7 | 67 | | 6 | 66 | | 8 (0.5) | 70 (0.6) |
| COMMON = 1 | 62 | 2 | | 63 | 2 | | 69 (0.8) | 2 (0.2) |
| COMMON = 0 | 38 | 98 | | 37 | 98 | | 31 (0.8) | 97 (0.2) |

Table S14 - Accuracy of predictors and proposed model in 10-fold Cross-Validation process, according to ClinVar version 2017-05-30. Mean and standard were calculated from ten steps of 10-fold Cross Validation.

| **Classifier** | **Accuracy**  **Mean**  **(± Std. Dev.)** | **Predictor=N,**  **Clinvar=0**  **Mean**  **(± Std. Dev.)** | **Predictor=P,**  **Clinvar=0**  **Mean**  **(± Std. Dev.)** | **Predictor=N,**  **Clinvar=1**  **Mean**  **(± Std. Dev.)** | **Predictor=P,**  **Clinvar=1**  **Mean**  **(± Std. Dev.)** |
| --- | --- | --- | --- | --- | --- |
| Extreme Gradient Boosting | 92 (0.0) | 91 (0.1) | 9 (0.1) | 7 (0.1) | 93 (0.1) |
| Ada Boost | 92 (0.1) | 92 (0.1) | 8 (0.1) | 8 (0.1) | 92 (0.1) |
| Bagging | 92 (0.0) | 90 (0.1) | 10 (0.1) | 8 (0.0) | 92 (0.0) |
| *** Proposed Tree** | 91 (0.1) | 91 (0.1) | 9 (0.1) | 9 (0.1) | 91 (0.1) |
| K Nearest Neighbors | 91 (0.1) | 90 (0.1) | 10 (0.1) | 7 (0.1) | 93 (0.1) |
| Random Forest | 91 (0.1) | 90 (0.1) | 10 (0.1) | 8 (0.1) | 92 (0.1) |
| Extra Trees | 91 (0.0) | 90 (0.1) | 10 (0.1) | 9 (0.1) | 91 (0.1) |
| SKLearn Decision Tree | 91 (0.1) | 90 (0.1) | 10 (0.1) | 9 (0.1) | 91 (0.1) |
| Extra Tree | 91 (0.0) | 90 (0.1) | 10 (0.1) | 9 (0.1) | 91 (0.1) |
| Multilayer Perceptron | 89 (0.0) | 89 (0.1) | 11 (0.1) | 11 (0.1) | 89 (0.1) |
| Logistic Regression | 89 (0.1) | 89 (0.1) | 11 (0.1) | 11 (0.1) | 89 (0.1) |
| Support Vector Machines (RBF kernel) | 89 (0.1) | 89 (0.1) | 11 (0.1) | 11 (0.1) | 89 (0.1) |
| Bernoulli Naive Bayes | 89 (0.0) | 88 (0.1) | 12 (0.1) | 10 (0.1) | 90 (0.1) |
| Support Vector Machines (Linear kernel) | 89 (0.0) | 89 (0.0) | 11 (0.0) | 11 (0.0) | 89 (0.0) |
| Gaussian Naive Bayes | 89 (0.0) | 88 (0.1) | 12 (0.1) | 10 (0.1) | 90 (0.1) |
| Linear Discriminant Analysis | 89 (0.0) | 90 (0.0) | 10 (0.0) | 12 (0.1) | 88 (0.1) |
| Quadratic Discriminant Analysis | 88 (0.1) | 92 (0.1) | 8 (0.1) | 14 (0.1) | 86 (0.1) |
| Nu-Support Vector Machines | 86 (0.1) | 85 (0.1) | 15 (0.1) | 14 (0.1) | 86 (0.1) |
| MetaSVM | 82 (0.1) | 75 (0.1) | 25 (0.1) | 11 (0.1) | 89 (0.1) |
| PROVEAN | 80 (0.1) | 78 (0.1) | 22 (0.1) | 18 (0.1) | 82 (0.1) |
| SIFT | 78 (0.1) | 81 (0.1) | 19 (0.1) | 24 (0.1) | 76 (0.1) |
| Polyphen | 77 (0.1) | 85 (0.1) | 15 (0.1) | 26 (0.1) | 74 (0.1) |

Table S15 - Coefficient φ for the two-by-two variable combinations of the proposed model.

|  | **SPP** | **ExAC** | **Ndamage** | **COMMON** |
| --- | --- | --- | --- | --- |
| **SPP** | 1.00 | 0.41 | 0.49 | -0.38 |
| **ExAC** | 0.41 | 1.00 | 0.55 | -0.77 |
| **Ndamage** | 0.49 | 0.55 | 1.00 | -0.48 |
| **COMMON** | -0.38 | -0.77 | -0.48 | 1.00 |


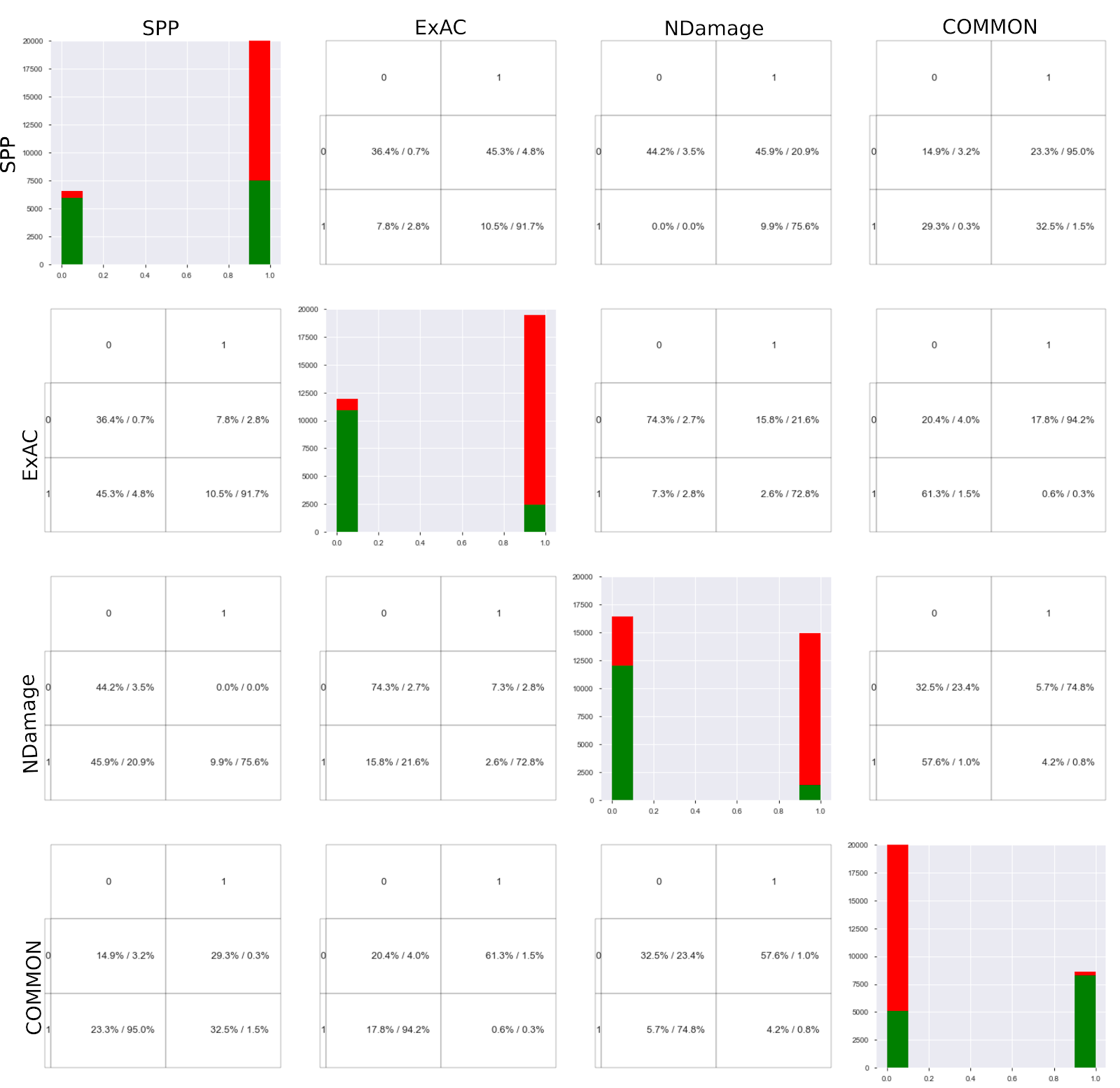


Figure S1 - Distribution of neutral and pathogenic mutations of variables used in proposed tree, according to ClinVar version 2017-05-30. Green bars indicate neutral mutations, and red bars, pathogenic ones. In tables’ columns and row names, 0 indicates the mutations classified by a variable as neutral, and 1, as pathogenic. In tables’ cells, numbers at left of the slash indicates the percentage of neutral mutations for each classification combination of each variables pair, and numbers at right of the slash, the percentage of pathogenic mutations.
